# A Non-Randomized Combined Program of Walking and Low-Load Resistance Exercise Improves Cognitive Function and Cardiometabolic Risk Factors in Community-Dwelling Elderly Women

**DOI:** 10.3390/healthcare10102106

**Published:** 2022-10-21

**Authors:** Jeonghyeon Kim, Seamon Kang, Haeryun Hong, Mingyu Joo, Hyunsik Kang

**Affiliations:** College of Sport Science, Sungkyunkwan University, Suwon 16419, Korea

**Keywords:** exercise intervention, cognitive decline, cardiometabolic risk factors, functional fitness

## Abstract

Background: This study examines whether changes in cardiometabolic risk factors, functional fitness, and depressive symptoms following a six-month exercise intervention were associated with cognitive function in Korean women aged 65 years and older. Methods: A non-randomized study design was used to compare post-intervention changes in measured variables between control (*n* = 30) and exercise (*n* = 30) groups. The exercise intervention consisted of three days of low-load resistance exercise and two days of walking. Cognitive function and depressive symptoms were assessed with the Korean version of the Mini–Mental State Examination and the Korean version of the Geriatric Depression Scale, respectively. Functional fitness was measured using a senior fitness test battery. Results: The exercise group showed a significant improvement in cognitive function (*p* < 0.001) in conjunction with significant decreases in blood glucose (*p* = 0.052), triglycerides (*p* = 0.011), insulin (*p* = 0.002), tumor necrosis factor-α (*p* = 0.043), and depressive symptoms (*p* = 0.006) and an increase in interleukin-10 (*p* = 0.037), compared with the control group. Multivariate stepwise regression showed that changes in depressive symptoms (*p* < 0.001), insulin resistance (*p* < 0.001), and upper body muscle strength (*p* = 0.003) were positively associated with cognitive function. Conclusion: A six-month exercise intervention consisting of walking and low-load/high-repetition elastic band resistance exercise has the potential to improve cognitive function, as well as physical function and cardiometabolic risk factors, and to decrease depressive symptoms in older women.

## 1. Introduction

Population aging is a global phenomenon by which the number and proportion of older adults are increasing worldwide. By 2050, adults aged 65 years and older will comprise approximately 17% of the world population, which is up from 9.1% in 2019 [1]. This global demographic transition portends a heavy burden on health and social systems because of age-associated health conditions (https://www.who.int/news-room/fact-sheets/detail/ageing-and-health/) (accessed on 10 September 2022). In particular, mental health conditions, including anxiety, depression, cognitive impairment, and dementia, have taken global priority through calls to action by governments and healthcare professionals due to disability and premature death worldwide [2].

Cognitive decline, ranging from mild cognitive impairment (MCI) to dementia, is a serious mental condition that commonly afflicts elderly people. Cognitive decline is sometimes considered an inevitable part of the aging process. However, the findings of previous studies suggest that cognitive decline with normal aging can be prevented or delayed [3]; one animal study even reversed it [4]. Therefore, developing a safe and effective strategy to protect cognitive function, such as physical activity and exercise, is an urgent need for all older adults, not just those who have MCI or dementia [5].

A substantial amount of evidence indicates that physical activity or exercise benefits metabolic and cardiovascular health in young, old, and healthy people, as well as patients with a variety of chronic conditions, by decreasing various risk factors and improving aerobic fitness [6]. Likewise, physical activity and exercise training have been used as nonpharmacologic methods for maintaining cognitive function and/or reducing cognitive decline among patients with MCI and dementia [7]. Previous studies have also suggested that cognitive decline with normal aging can be prevented or delayed via nonpharmacologic strategies [3]. In contrast to numerous studies of patients with MCI or dementia, however, only a few evidence-based studies support the cognitive benefits of physical activity and exercise for healthy older adults. Most previous studies have focused on short-term effects and have not found that exercise intervention in older adults has cognitive benefits [8,9], although some long-term studies have found cognitive benefits [10,11].

The lack of evidence about exercise interventions has multifactorial causes, including exercise intensity inadequate to promote aerobic fitness, poor exercise adherence, short study duration, low statistical power, and the use of clinically irrelevant measures [12]. In addition, although there is a linear relationship between training load and an increase in muscle size and strength in older women [13], high-load resistance training often increases the risk of injury or overuse [14] and impairs post-exercise physical function [15] in elderly persons. Strength-naïve women may exhibit superior adherence to low-load elastic band training compared with stationary resistance training programs [16] and experience superior cognitive benefits compared with high-load resistance exercise [17]. Elastic band training provides more benefits compared with free-weight training, such as reduced injury risk and enhanced functional strength [18].

Nonetheless, there is not enough evidence to say that the exercise interventions applicable to elderly people living independently in the community effectively delay or prevent cognitive decline with normal aging [8]. This study aims to investigate the cognitive benefits of a long-term exercise intervention consisting of walking and low-load and high-repetition resistance exercise for elderly community-dwelling Korean adults.

## 2. Materials and Methods

### 2.1. Participants and Study Design

**Figure 1:**
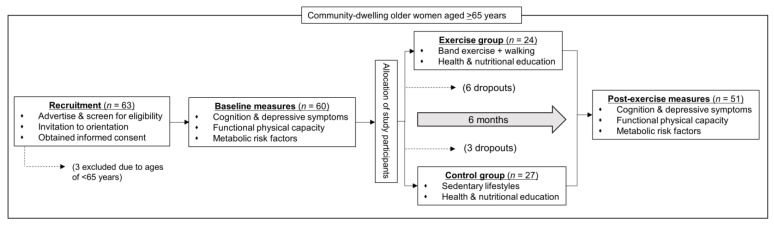
Illustration of overall study design and measurement procedure.

Figure 1 represents the overall study design. Briefly, 63 women aged 65 years and older were recruited via flyers at local community centers (Suwon, Korea). The inclusion criteria were healthy, community-dwelling women aged 65 years and older with: (a) no diagnosed mental disorders based on their health record, including depression, cognitive impairments, and dementia, (b) no moderate or severe pain in the knee on most days of the month, (c) no difficulty with most activities of daily living due to knee pain, and (d) no regular participation in any formal exercise program during the previous 6 months. The exclusion criteria were: (a) the presence of a medical condition that precluded participation in a safe exercise program, (b) inflammatory arthritis, (c) regular participation in exercise (more than twice a week for at least 20 min), and (d) inability to walk without assistance.

All participants were assessed in the pre-intervention period and at the end of the intervention. During the pre-intervention period, all the initial volunteers were invited to an orientation session and assessed for inclusion and exclusion criteria. Three persons were excluded due to age (younger than 65 years and older), and the remaining 60 women, who were all apparently healthy, signed written informed consent forms and underwent baseline measurements.

This non-randomized study investigated the effects of a six-month intervention consisting of walking and elastic band resistance exercise on functional physical capacity, cardiometabolic risk factors, depression, and cognitive function using control and exercise groups. Following the baseline measurements, all the participants were assigned to either the exercise intervention group (*n* = 30) or the control group (*n* = 30) based on their date of birth and preference. The sample size of each group was calculated using G*Power software (version 3.1.9.7; Heinrich-Heine-Universität Düsseldorf, Düsseldorf, Germany) to detect a significant difference in the primary outcome of cognitive function between the control (*n* = 25) and intervention (*n* = 25) groups, with a statistical power of 85% and a probability of alpha error of 0.05.

The research staff were not blinded to participant group allocation. The intervention group participated in a six-month intervention consisting of an exercise program (5 days per week with 3 days of resistance exercise and 2 days of walking) and health education (2 days per week), whereas the control group participated only in the health education sessions (2 days per week). After the six-month intervention, 51 participants (27 in the control group and 24 in the exercise group) underwent post-intervention measurements consisting of the same procedures used for the baseline measurements. The efficacy of the intervention was evaluated by comparing post-intervention changes in the measured parameters between the groups.

### 2.2. Exercise Intervention

The exercise intervention consisted of 3 days (i.e., Monday, Wednesday, and Friday) of low-load and high-repetition elastic band resistance exercise and 2 days (i.e., Tuesday and Thursday) of walking for 6 months under the supervision of certified exercise leaders. Resistance exercise using a low-load elastic band (TheraBand, Performance Health, Akron, OH, USA) was individually designed to stimulate all the major muscle groups—chest press, leg press and extension, shoulder press, abdominal curl, and biceps curl. Resistance exercise consisted of two sets of 15–20 repetitions for each muscle group. Walking, with a duration of 20 min per session and an intensity of 40–50% of heart rate reserve, was performed on a walking trail in a public park under the supervision of certified exercise leaders. During each session, the participants wore heart monitors (Polar OH1 HR Sensor, Polar, Finland) to track the prescribed exercise intensity. Additionally, both the intervention and control groups received health education sessions (twice per month) from a nurse on issues related to arthritis management, health updates, and social support.

### 2.3. Measurements

Outcome measurements were performed by a trained research team, consisting of a research coordinator and two certified exercise specialists, at baseline and after the six-month intervention. Additionally, a certified nurse trained in cognitive assessments conducted face-to-face interviews to measure the primary outcome of cognitive function and the secondary outcomes of depressive symptoms, functional physical capacity, and cardiometabolic risk factors.

#### 2.3.1. Cognitive Function

Cognitive function was assessed using the Korean version of the Mini–Mental State Examination (K-MMSE), optimized for screening dementia (MMSE-DS). The MMSE-DS is an updated and validated version of the Korean version of the MMSE (MMSE-KC), included in both the Consortium to Establish a Registry for Alzheimer’s Disease Assessment Packet and the Seoul Neuropsychological Screening Battery. Scores range from 0 to 30, with higher scores indicating better cognition [19].

#### 2.3.2. Depressive Symptoms

Depression was assessed using the Korean version of the 15-item Geriatric Depression Scale (SGDS-K). The SGDS comprises 15 dichotomous depression items, and the scores range from 0 to 15, with higher scores indicating more severe depressive symptoms. The SGDS-K has been validated in the Korean elderly population [20].

#### 2.3.3. Functional Physical Capacity

Functional physical capacity was evaluated with a senior fitness test (SFT) battery, as described previously [21]. Briefly, the SFT assesses the physiological capacity to perform normal daily activities independently and safely without the appearance of fatigue. After a 10 min of warm-up (i.e., walking around an indoor track and stretching), participants performed the SFT in the following order: (1) chair stand for 30 s to assess lower body strength (number of stands); (2) arm curl for 30 s to assess upper body strength (number of curls); (3) chair-sit-and-reach to assess the flexibility of the lower extremities (cm); (4) the 2.44-m up-and-go to assess agility as an index of basic mobility skill (seconds); and (5) a 6-min walk test to assess aerobic capacity (meters). The test validity and reliability of the SFT were previously published [21].

#### 2.3.4. Cardiometabolic Risk Factors and Serum Cytokines

Weight (kg) and height (cm) were measured with a stadiometer attached to a scale (Jenix, Seoul, Korea). Body mass index (kg/m^2^) was calculated by dividing weight (kg) by the square of height (m^2^). Systolic and diastolic blood pressure levels were measured. Blood samples were collected from an antecubital vein at 07:00–08:00 a.m. following an overnight fast, allowed to stand at room temperature for 30 min, and centrifuged at 3000 rpm for 10 min. Then, aliquots of the serum samples were stored at −80 °C until analysis.

Glucose, triglycerides (TG), total cholesterol (TC), high-density lipoprotein cholesterol (HDLC), and low-density lipoprotein cholesterol (LDLC) were measured using enzymatic calorimetric tests with a Beckman Coulter analyzer (AU680, Brea, California, USA). Insulin was measured using an enzyme-linked immunosorbent assay (ALPCO, Salem, New Hampshire, USA) and a microplate reader (Labsystems Multiskan MS 352; Thermo Fisher Scientific, Waltham, MA, USA). The intra- and inter-assay coefficients of variation were <5% and <6%, respectively. The homeostasis assessment model for insulin resistance (HOMA-IR) was calculated using the formula HOMA-IR = glucose (mg/dL) × insulin (μU/mL)/405.

Serum cytokines were measured using enzyme-linked immunosorbent assay kits (ALPCO, Salem, NH, USA) and a microplate reader (Labsystems Multiskan MS 352; Thermo Fisher Scientific, Waltham, MA, USA). The samples were prepared in duplicate, and average values were recorded for statistical analyses. Intra- and inter-assay coefficients of variation were <7% and <12%, respectively. The cytokines we tested can be grouped as pro-inflammatory cytokines (interleukin (IL)-1β, IL-6, and tumor necrosis factor α (TNF-α)) and anti-inflammatory cytokines (IL-4 and IL-10).

### 2.4. Statistics

Data are presented as means and standard deviations. Prior to the statistical analyses, the data were visually checked for normality with QQ-plots and histograms. For baseline measurements, independent t-tests were used to compare between-group differences in measured variables. Two-way ANOVA with repeated measures was used to compare between-group changes in measured variables from 0 to 6 months. Multivariate stepwise regression was used to estimate significant determinants in changed scores for cognitive function from 0 to 6 months. Statistical significance was tested at α = 0.05 using SPSS-PC version 27.0 (IBM SPSS Statistics, Armonk, New York, USA).

## 3. Results

Table 1 presents descriptive statistics for the study participants at baseline. Their mean age was 72.9 ± 4.7 years, and they had an average BMI of 20.9 ± 4.4 kg/m^2^. The control and exercise groups did not differ significantly in any of the baseline measurements (Table 1).

**Figure 2:**
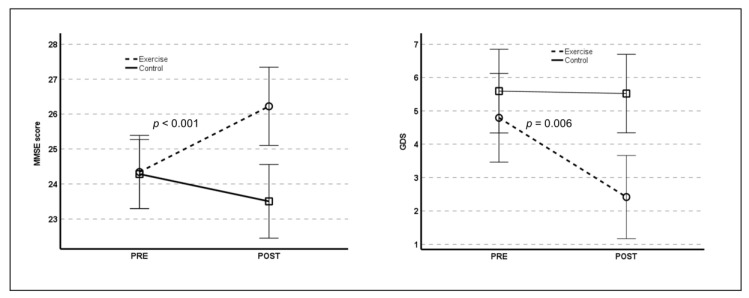
Time-by-group interactions for cognitive function (**left**) and depressive symptoms (**right**). MMSE: Mini–Mental state examination; GDS: geriatric depression scale.

Significant time-by-group interactions were found for changes in cognitive function and depressive symptoms. As shown in Figure 2, the exercise group showed a significant post-intervention increase in cognitive function (F_(1,49)_ = 34.486, *p* < 0.001) and a significant post-intervention decrease in depressive symptoms (F_(1,49)_ = 8.169, *p* = 0.006) compared with the control group.

**Figure 3:**
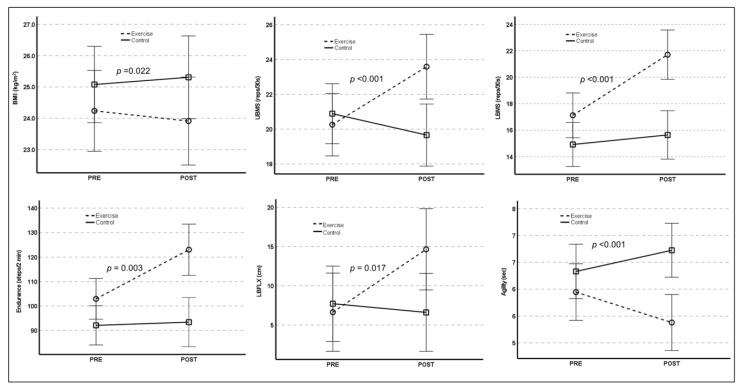
Illustration of time-by-group interactions for body mass index and physical functional capacity. BMI: body mass index; UBMS: upper body muscle strength; LBMS: lower body muscle strength; LBFLX: lower body flexibility.

Significant time-by-group interactions were found for changes in several parameters of physical functional capacity. As shown in Figure 3, the exercise group showed significant post-intervention improvements in upper body muscle strength (UBMS; F_(1,49)_ = 17,478, *p* < 0.001), lower body muscle strength (F_(1,49)_ = 17,014, *p* < 0.001), endurance (F_(1,49)_ = 9.885, *p* = 0.003), lower body flexibility (F_(1,49)_ = 6.137, *p* = 0.017), and agility (F_(1,49)_ = 6.325, *p* < 0.001) compared with the control group. No significant time-by-group interaction was found in upper body flexibility (F_(1,49)_ = 3492, *p* = 0.518).

**Figure 4:**
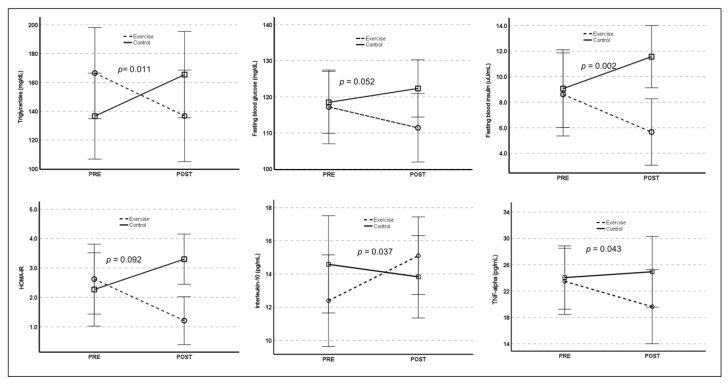
Illustration of time-by-group interactions for metabolic risk factors and serum cytokines.

Significant time-by-group interactions were found for changes in several cardiometabolic risk factors and cytokines. As shown in Figure 4, the exercise group showed significant post-intervention improvements in fasting blood glucose (F_(1,49)_ = 3.982, *p* = 0.052), TG (F_(1,49)_ = 8.670, *p* = 0.011), insulin level (F_(1,49)_ = 10.484, *p* = 0.002), and HOMA-IR (F_(1,49)_ = 7.547, *p* = 0.009) in conjunction with significant decreases in BMI (F_(1,49)_ = 5.557, *p* = 0.022) and TNF-α (F_(1,49)_ = 4.353, *p* = 0.043) and an increase in IL-10 (F_(1,49)_ = 4.612, *p* = 0.037) compared with the control group. No significant time-by-group interactions were observed in resting blood pressure, TC (F_(1,49)_ = 3.195, *p* = 0.080), LDL-C (F_(1,49)_ = 1.134, *p* = 0.292), HDL-C (F_(1,49)_ = 0.023, *p* = 0.879), IL-6 (F_(1,49)_ = 1.035, *p* = 0.314), IL-1β (F_(1,49)_ = 0.197, *p* = 0.659), or IL-4 (F_(1,49)_ = 1.229, *p* = 0.277).

**Figure 5:**
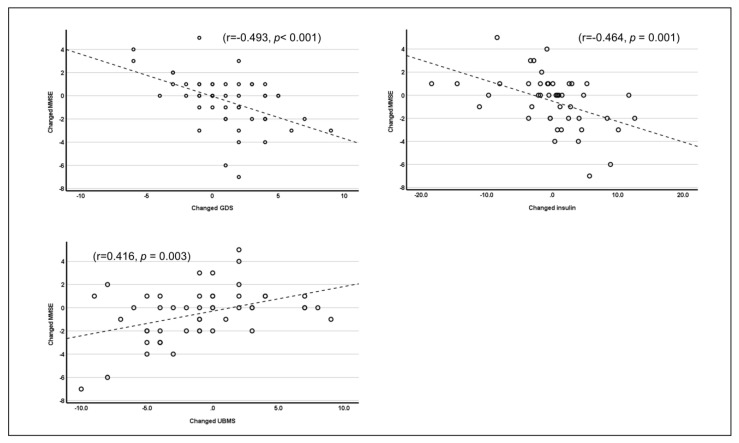
The relationships between changes in cognitive function, depressive symptoms, insulin, and upper body muscle strength (UBMS) in the total group.

Table 2 summarizes the results of the stepwise multiple regression analyses for pre-to-post-intervention changes in cognitive function in the total study population. Changes in depressive symptoms (*p* < 0.001), insulin resistance (*p* < 0.001), and UBMS (*p* = 0.003) were significant and independent predictors of change in cognitive function. As shown in Figure 5, an increase in cognitive function was significantly associated with decreases in depressive symptoms (r = 0.493, *p* < 0.001) and insulin resistance (r = -0.464, *p* < 0.001) and an increase in UBMS (r = 0.416, *p* = 0.003).

## 4. Discussion

In this study, we examined whether changes in cardiometabolic risk factors, functional fitness, and depressive symptoms following a six-month exercise intervention were associated with changes in cognitive function in older Korean women who were voluntarily recruited from the local community. Our findings show that the exercise intervention (walking and low-load, high-repetition elastic band resistance exercise) resulted in an improvement in cognitive function, functional physical capacity, and cardiometabolic risk factors, as well as decreases in depressive symptoms and serum proinflammatory cytokines.

To the best of our knowledge, we are the first to report a significant association between an improvement in cognitive function and improvements in depressive symptoms, insulin resistance, and muscular strength following a combined exercise intervention. Furthermore, we found no exercise-related adverse events, an average retention rate of 85%, and average exercise compliance of 80%, implying that the exercise intervention of walking and elastic band resistance exercise was a safe and effective nonpharmacologic strategy that can be applied to elderly people.

### 4.1. Exercise Intervention and Functional Physical Capacity

The six-month exercise intervention consisting of walking and low-load, high-repetition elastic band resistance exercise produced significant increases in upper and lower body muscle strength, endurance, lower body flexibility, and agility, though upper body flexibility did not change significantly. These findings are consistent with the findings of previous studies involving older adults and patients with chronic diseases such as diabetes.

In a systematic review and meta-analysis involving 2608 community-dwelling older adults from 28 studies, for example, Chase et al. [22] found that the beneficial effects of exercise interventions for physical functioning occurred in a dose–response manner and that such interventions were especially effective among frail subjects. Similarly, the benefits of structured exercise for functional capacity have been reported in previous studies involving community-dwelling elderly people [23]. Martínez-Velilla et al. [24] conducted a randomized controlled clinical trial to examine the effects of an exercise intervention on functional decline in 370 very elderly patients during acute hospitalization and found that, at discharge, the exercise intervention group had better functional independence, cognitive function, and quality of life than the control group. Additionally, Kwak, Kim, and Lee [25] showed that eight weeks of elastic band resistance exercise improved balance, mobility, gait function, flexibility, and fall efficacy in 45 outpatients in South Korea.

Taken together, along with walking, resistance exercise with elastic bands is safe and feasible for the promotion of physical and mental health in strength-naïve elderly women. The findings of the current and previous studies suggest that low-load and high-repetition elastic band resistance exercises can be used as an alternative to traditional free weights or stationary resistance exercise training for elderly persons.

### 4.2. Exercise Intervention and Cardiometabolic Risk Factors and Serum Cytokines

Our six-month exercise intervention also resulted in improvements in fasting glucose, insulin level, TG, and HOMA-IR, in conjunction with more favorable cytokine profiles. Metabolic syndrome, or insulin resistance syndrome, is a well-known risk of cognitive decline, including MCI and dementia. For example, Wang et al. [26] conducted a cross-sectional study of 5854 community-dwelling participants and showed that metabolic syndrome risk factors such as abdominal obesity and elevated blood pressure were significantly associated with an increased risk of cognitive impairment. In a systematic review and meta-analysis involving 20 studies (10 for adults and 10 for adolescents), Yates et al. [27] examined whether metabolic syndrome was negatively associated with cognitive function and brain imaging. They found that metabolic syndrome could have negative effects on cognition and brain structure, perhaps via insulin resistance-associated impairments in vascular reactivity, neuroinflammation, oxidative stress, tau hyperphosphorylation, abnormal amyloid-beta metabolism, or abnormal brain lipid metabolism [28].

Our current findings are consistent with findings from previous population-based studies which report that physical activity is associated with a lower risk of metabolic syndrome and improvements in insulin sensitivity and metabolic risk factors [29]. In a randomized controlled trial involving 115 men and women aged 55 to 75 years, Stewart et al. [30] examined the effects of a six-month exercise program on risk factors associated with metabolic syndrome. In that study, they showed that the exercise program resulted in improved aerobic fitness in conjunction with favorable changes in risk factors for cardiovascular disease and diabetes, independent of changes in fitness or body weight. The effects of exercise training as a primary countermeasure against age-related chronic disease, mobility disability, cardiovascular disease, type 2 diabetes, and cancer, are well summarized in a review study [31]. In addition to favorable changes in cardiometabolic risk factors, it is consistently reported that physical activity and exercise training contribute to brain health, including cognitive function, by enhancing anti-inflammatory and anti-oxidative responses in the brain, neurogenesis, and neuronal plasticity in older adults [32]. Physical activity and exercise, when combined with pharmacologic treatments, might also reduce cognitive decline and dementia risk at any age [33].

### 4.3. Exercise Intervention and Mental Health

Our six-month exercise intervention improved mental health, as shown by the improvement in cognitive function and the decrease in depressive symptoms. The antidepressant effect of physical activity, including regular exercise, has been consistently reported in previous studies involving older adults [34] and depressive patients [35], and it presumably works by stimulating adult neurogenesis or increasing gray matter volume in the hippocampus, prefrontal cortex, and anterior cingulate cortex [36].

The cognitive benefit of the exercise intervention in this study is consistent with the findings of previous studies. In a systematic review and meta-analysis of 33 published studies, for example, Northey et al. [37] examined the effects of different exercise interventions on cognitive function in community-dwelling adult adults aged 50 years and older. They concluded that, regardless of modality, any exercise program with a duration of 45–60 min per session and at least moderate intensity significantly improved cognitive function. In a systematic review involving 11 randomized, non-randomized, and uncontrolled trials, Pattern et al. [38] examined the effects of exercise interventions on mental health and health-related quality of life in women with poly ovary syndrome. In that study, they showed that exercise training improved health-related quality of life in all 11 trials, and half of the 11 studies reported significant improvements in depression and anxiety symptoms. Taken together, the findings from the current and previous studies support the potential use of exercise intervention as a safe and effective strategy for improving mental health, including depression symptoms and cognitive function, in community-dwelling older women.

### 4.4. Potential Explanations for the Cognitive Benefit of Exercise Intervention

Multivariate stepwise regression showed that changes in insulin resistance, upper body muscle strength, and depressive symptoms following the six-month intervention were significant determinants of changes in cognitive function. The cognitive benefit of this long-term exercise intervention can be explained in several ways.

First, an exercise intervention could positively contribute to cognitive function via its antidepressant effect, although the underlying mechanisms of the antidepressant effect remain to be unveiled. Second, exercise could positively contribute to cognitive function via improvements in cerebral perfusion, blood pressure, amyloid clearance, and other factors [39]. Third, sarcopenia, defined as loss of muscle strength or function, is associated with a high risk of cognitive impairment in older adults [40]. In contrast, physical activity and exercise training have been found to upregulate the expression of neurotrophic factors such as brain-derived nerve growth factor and insulin-like growth factor-1, which could contribute positively to cognitive function by enhancing neurogenesis and neural plasticity [41]. Fourth, excessive inflammation and oxidative stress are associated with age-related cognitive decline. In contrast, physical activity and muscular strength are associated with decreased inflammation and oxidation, along with increased anti-inflammatory and anti-oxidative factors in the brain, contributing to brain health, including cognitive function, via enhanced neuronal survival and neurogenesis [42].

### 4.5. Study Limitations

This study has some limitations. First is the risk of selection bias due to the quasi-randomization used in participant allocation. Randomized controlled trials are needed to confirm the outcomes of this study. Second, this study included only women. Previous studies reported gender differences in the effectiveness of exercise interventions. The current findings need to be confirmed in older men for generalization. Third, we did not control other lifestyle factors, including dietary intake, smoking, alcohol consumption, and others. We cannot rule out the possibility that such external factors influenced the outcomes of this study. Caution is needed when interpreting the outcomes of this study.

## 5. Conclusions

In this study, we found that a long-term exercise program consisting of walking and low-load and high-repetition resistance exercise is safe and feasible for community-dwelling older adults. In addition, we found that the exercise intervention improved cognitive function, cardiometabolic risk factors, serum cytokines, and depressive symptoms in this study population. Furthermore, we showed that an improvement in cognitive function was significantly associated with improvements in depressive symptoms, insulin resistance, and muscular strength following an exercise intervention. These findings suggest that a structured exercise program can be recommended as a nonpharmacologic strategy for elderly people with or without cognitive decline to improve their physical fitness and metabolic profiles and mitigate cognitive decline with normal aging.

## Figures and Tables

**Table 1:** Physical characteristics of study participants at baseline.

	Control (*n* = 30)	Exercise (*n* = 30)	Total (*n* = 60)	*p*-Value
Age (years)	73.3 ± 4.9	72.7 ± 4.2	73.0 ± 4.5	0.612
Body mass index (kg/m^2^)	24.7 ± 3.2	24.2 ± 3.1	24.5 ± 3.1	0.485
Systolic BP (mmHg)	128.4 ± 13.4	129.5 ± 10.2	128.9 ± 11.8	0.730
Diastolic BP (mmHg)	73.8 ± 9.3	71.1 ± 8.1	72.5 ± 8.7	0.235
FBG (mg/dL)	117.5 ± 21.3	118.5 ± 22.0	118.0 ± 21.5	0.868
TC (mg/dL)	166.4 ± 35.9	186.6 ± 42.8	176.5 ± 40.5	0.052
TG (mg/dL)	129.7 ± 86.0	145.5 ± 69.3	137.6 ± 77.8	0.435
HDLC (mg/dL)	49.8 ± 13.4	54.1 ± 12.1	51.9 ± 12.8	0.197
LDLC (mg/dL)	94.0 ± 28.8	102.9 ± 37.2	98.4 ± 33.3	0.308
Insulin (μU/mL)	8.9 ± 7.7	9.4 ± 8.6	9.2 ± 8.1	0.813
HOMA-IR	2.2 ± 2.0	2.5 ± 2.8	2.4 ± 2.5	0.602
IL-4 (pg/mL)	5.5 ± 2.9	6.6 ± 2.0	6.1 ± 2.5	0.485
IL-10 (pg/mL)	7.4 ± 3.1	9.6 ± 4.1	8.4 ± 3.5	0.280
IL-6 (pg/mL)	5.6 ± 2.6	6.5 ± 3.9	6.1 ± 3.3	0.362
IL-1beta (pg/mL)	2.3 ± 2.1	2.5 ± 2.0	2.4 ± 2.0	0.726
TNF-α (ng/mL)	23.7 ± 14.3	23.1 ± 7.9	23.4 ± 11.6	0.912
UBMS (reps/30 s)	21.4 ± 4.6	20.3 ± 4.7	20.8 ± 4.6	0.330
LBMS (reps/30 s)	15.8 ± 5.8	16.7 ± 4.4	16.3 ± 5.1	0.500
Endurance (steps/2 min)	94.0 ± 23.8	102.5 ± 18.4	98.2 ± 21.5	0.129
UBFLX (cm)	−12.1 ± 11.0	−13.3 ± 9.7	−12.7 ± 10.3	0.657
LBFLX (cm)	9.6 ± 12.8	4.3 ± 10.3	6.9 ± 11.8	0.080
Agility (sec)	6.2 ± 1.6	5.8 ± 1.2	6.0 ± 1.4	0.284
GDS (score)	5.6 ± 3.8	4.7 ± 2.8	5.2 ± 3.4	0.306
MMSE (score)	24.3 ± 4.3	25.0 ± 2.0	24.7 ± 3.4	0.382

BP: blood pressure; FBG: fasting blood glucose; TC: total cholesterol; TG: triglycerides; HDLC: high-density lipoprotein cholesterol; LDLC: low-density lipoprotein cholesterol; HOMA-IR: homeostasis assessment model for insulin resistance; UBMS: upper body muscle strength; LBMS: lower body muscle strength; UBFLX: upper body flexibility; LBFLX: lower body flexibility; IL: interleukin; TNF: tumor necrosis factor; MMSE: Mini–Mental state examination; GDS: geriatric depression scale.

**Table 2:** Determination of predictors for changed cognitive function after an exercise intervention.

Predictors	Unstandardized β	SE	t	95% CI	*p*-Value
ΔGDS	−0.342	0.073	−4.659	−0.491~−0.190	<0.001
ΔInsulin	−0.185	0.044	−4.205	−0.275~−0.094	<0.001
ΔUBMS	0.214	0.066	3.257	0.079~0.350	0.003

CI: confidence interval; SE: standard error; GDS: geriatric depression scale; UBMS: upper body muscle strength.

## Data Availability

The datasets used and analyzed during this study are available from the corresponding author upon reasonable request.
